# An analysis of health system resources in relation to pandemic response capacity in the Greater Mekong Subregion

**DOI:** 10.1186/1476-072X-11-53

**Published:** 2012-12-14

**Authors:** Piya Hanvoravongchai, Irwin Chavez, James W Rudge, Sok Touch, Weerasak Putthasri, Pham Ngoc Chau, Bounlay Phommasack, Pratap Singhasivanon, Richard Coker

**Affiliations:** 1 Faculty of Medicine, Chulalongkorn University, Bangkok, 10330, Thailand; 2 Faculty of Tropical Medicine, Mahidol University, Bangkok, 10400, Thailand; 3 Communicable Diseases Policy Research Group, London School of Hygiene & Tropical Medicine, 9th Floor, Satharanasukwisit Building, Mahidol University, 420/1 Rajwithi Road, Ratchathewi, Bangkok, 10400, Thailand; 4 Cambodia Ministry of Health, Department of Communicable Disease Control, 151-153 Kampuchea Ground Avenue, Phnom Penh, Cambodia; 5 International Health Policy Programme-Thailand, Ministry of Public Health, Nonthaburi, 11000, Thailand; 6 Vietnam Military Medical University, Hanoi, Vietnam; 7 National Emerging Infectious Diseases Coordination Office, Fa Ngoum Road, Vientiane, Laos

**Keywords:** Health system, Pandemic influenza, Health equity, Resource mapping, Resource allocation, Antivirals, Southeast Asia

## Abstract

**Background:**

There is increasing perception that countries cannot work in isolation to militate against the threat of pandemic influenza. In the Greater Mekong Subregion (GMS) of Asia, high socio-economic diversity and fertile conditions for the emergence and spread of infectious diseases underscore the importance of transnational cooperation. Investigation of healthcare resource distribution and inequalities can help determine the need for, and inform decisions regarding, resource sharing and mobilisation.

**Methods:**

We collected data on healthcare resources deemed important for responding to pandemic influenza through surveys of hospitals and district health offices across four countries of the GMS (Cambodia, Lao PDR, Thailand, Vietnam). Focusing on four key resource types (oseltamivir, hospital beds, ventilators, and health workers), we mapped and analysed resource distributions at province level to identify relative shortages, mismatches, and clustering of resources. We analysed inequalities in resource distribution using the Gini coefficient and Theil index.

**Results:**

Three quarters of the Cambodian population and two thirds of the Laotian population live in relatively underserved provinces (those with resource densities in the lowest quintile across the region) in relation to health workers, ventilators, and hospital beds. More than a quarter of the Thai population is relatively underserved for health workers and oseltamivir. Approximately one fifth of the Vietnamese population is underserved for beds and ventilators. All Cambodian provinces are underserved for at least one resource. In Lao PDR, 11 percent of the population is underserved by all four resource items. Of the four resources, ventilators and oseltamivir were most unequally distributed. Cambodia generally showed higher levels of inequalities in resource distribution compared to other countries. Decomposition of the Theil index suggests that inequalities result principally from differences within, rather than between, countries.

**Conclusions:**

There is considerable heterogeneity in healthcare resource distribution within and across countries of the GMS. Most inequalities result from within countries. Given the inequalities, mismatches, and clustering of resources observed here, resource sharing and mobilization in a pandemic scenario could be crucial for more effective and equitable use of the resources that are available in the GMS.

## Background

The Southeast Asia region plays a key role in the global circulation and long-term evolution of influenza viruses [1], and is considered likely to be at the epicentre for the emergence of a novel influenza strain with pandemic potential [2]. The ability of the region to mitigate the consequences of the emergence of a pandemic virus is of profound importance [3-5]. There is an increasing perception that countries cannot work in isolation to protect themselves from emerging pathogens and that international cooperation is essential [4,6-11]. To differing degrees, the Association of Southeast Asian Nations (ASEAN), the Ayeyawady-Chao Phraya-Mekong Economic Cooperation Strategy, and the Asia-Pacific Economic Cooperation forum have all endorsed transnational cooperative activities with the support of WHO South East Asia and Western Pacific Regional Offices [12]. Examples of cooperative activities include information and experience sharing, development of guidelines and toolkits, and the design and implementation of pandemic preparedness simulation exercises [12]. At the forefront of cooperative activities, the Mekong Basin Disease Surveillance (MBDS) network has been facilitating cooperation in surveillance and control efforts across the land borders of countries in the Greater Mekong Subregion (GMS; specifically, Cambodia, Lao PDR, Myanmar, Thailand, Vietnam, and Yunnan and Guangxi provinces of China) for more than a decade [2,12].

However, while surveillance and outbreak response activities have been strengthened in Southeast Asia through such initiatives, relatively little attention has been given to investing in, and sharing of, healthcare resources for pandemic mitigation. Recent experience from pandemic influenza A (H1N1)-2009 highlights how health system capacities, even in developed countries, can be stretched by relatively mild pandemic scenarios [13-15]. Given the wide variation in socio-economic conditions in the GMS, and the aforementioned initiatives for increased international cooperation, resource mobilisation and sharing could play a particularly important role in a pandemic response in this region [16].

The need and potential for resource sharing across administrative boundaries for pandemic mitigation will depend at least partly on how healthcare resources are currently distributed, both within and between countries, in the region. Therefore, we conducted an analysis of healthcare resources across four countries in the GMS of Southeast Asia that share land borders: Cambodia, Lao PDR, Thailand and Vietnam. Health system resource data collected across the four countries were analysed to quantitatively estimate and compare the inequalities, clustering, and mismatches of resources within and between countries. This analysis is a part of a larger portfolio of work, the Asia*FluCap* project, which aims to evaluate health system capacity in Southeast Asia in response to different pandemic influenza scenarios.

## Methods

### Data collection on key resources

Data were collected using specific questionnaires designed by the AsiaFluCap Project. Quantities of resource items were enumerated through questionnaires administered to hospitals, district health offices, and ministries of health at central level, in each of the four study countries during March to September 2009. Hospital and district level questionnaires were administered nationwide in all countries.

The hospital and district questionnaires included two sections: the first section collected basic information on the district or hospital and the respondent; the second section assessed resource availability in the health facility (hospital questionnaire) or elsewhere in the district (district questionnaire). It was emphasized that district questionnaires only include data on resource availability outside hospital settings to avoid double counting. Through these questionnaires, data were collected on a total of 57 resource items across the following six categories: (I) health care facilities and their bed capacities, (II) human resources, (III) equipment and machines, (IV) personal protective equipments (PPE) and drugs, (V) laboratory and investigation capacity, and (VI) access to communication technology (see Additional file 1 for a full list of resources). The definitions and sample pictures of all resource items were provided on the back of the questionnaires to ensure common understanding of the type of the resource. The questionnaires were translated into national language with reverse translation to ensure consistency across languages.

### Extrapolation of missing data

Missing data occurred from non-responses or incomplete answers in the district or hospital questionnaires. We therefore use linear prediction models to extrapolate these missing values based on a number of district characteristics such as total number of hospital beds or public hospital beds, population size, geographic location (region/province). The extrapolation exercise was done separately for each resource and for each country to obtain best model fit. For oseltamivir and ventilators, two-step models were utilized to first estimate the likelihood of having any oseltamivir or ventilators and then to predict the number of units. Further details are provided in the Additional file 1: Text S1 and Table S2. All analyses for data extrapolation were done using STATA.

### Data analysis

For this comparative analysis we focus on four key health system resources: antiviral drugs (specifically, oseltamivir), hospital beds, mechanical ventilators and healthcare workers (doctors and nurses). These items were chosen because of their critical importance in responding to pandemic influenza. Descriptive analysis was carried out to estimate the availability and densities (per population) by geographical location. The geographical distribution was analysed at province level in all countries.

Identification of regionally underserved and oversupplied areas was carried out using an operational definition as follows. We define relatively underserved areas for each type of resource as the provinces which have a resource density within the lowest quintile of all resource densities across provinces in the four GMS study countries. Areas with multiple under-service provision are provinces relatively underserved with more than one of the four resource items. Areas relatively oversupplied with certain resource items are provinces with a resource density within the top quintile based on resource densities across provinces in the four GMS countries. Similarly, areas with multiple over-service provision are provinces with a relative oversupply of more than one resource item. In addition, we identify nationally underserved and oversupplied areas in each country based on lowest and highest quintiles, respectively, of resource densities across provinces within each country.

We define areas with a resource mismatch as provinces that are both relatively underserved for at least one resource type and relatively oversupplied for at least one other resource type. For example, a province relatively oversupplied for beds but relatively underserved for the health workforce is considered a province with a resource mismatch.

Inequalities in the distribution of resources across the GMS region were comparatively assessed using two commonly used composite inequality indices: Gini coefficient and Theil index [17-19]. These two indices were calculated based on province level resource densities with population weighting. Methods for calculating these indices have been described elsewhere [20-22]. Since calculation of the Theil index involves logarithmic transformation of the ratio of resource share to population share, it cannot be computed when there are zero values in the dataset. Therefore, since some resources (specifically, oseltamivir and ventilators) were absent from some provinces, a small constant value of 10^-10^ was added to all resource data across all provinces prior to calculating the Theil index. (For resources for which there were no zero values, specifically beds and health workers, Theil values were not affected by this adjustment, indicating its neutrality). Decomposition analysis of the Theil index, to identify within-country and between-country components of inequality, was also conducted using ineqdec0 package [23] under STATA software.

We identified clustering or dispersion of these key resources in the four GMS countries by geographical information system (GIS) analysis using GeoDA 0.95i software. Clustering or dispersion of resources is defined in relation to a province in which the availability (per capita) of a specific resource is significantly similar or different from its neighboring provinces (spatial autocorrelation). Spatial autocorrelation was estimated using Moran’s index [24], and considers correlation between resource availability per capita in each province and average resource availability per capita surrounding each province. Positive spatial autocorrelation represents spatial clustering, i.e., clustering of areas with similar densities, in which “high-high” and “low-low” clusters define a group of adjacent provinces with similarly high and low resource densities, respectively, relative to other provinces in the region. Negative spatial autocorrelation represents spatial outliers, i.e., clustering of areas with different densities, in which a “high-low” cluster defines a province with relatively high resource density surrounded by provinces with relatively low resource densities, and a “low-high” cluster defines the converse pattern. Neighboring provinces are identified as areas that share a common border with the province.

Clustering or dispersion of resources was assessed using Moran’s Index for global spatial autocorrelation. Local clusters were then identified using the Local Indicators of Spatial Autocorrelation (LISA) with the Empirical Bayes adjustment. The spatial weights were constructed using the rook case considering only adjacent areas as neighbors (first order contiguity). The level of significance was set at *p* < 0.05 and simulation runs to 9,999.

Geographical maps of resource availability, relative shortage, multiple shortages, resource mismatches, and clustering of key resources were created using ArcGIS software.

## Results

The response rates for district and hospital questionnaires in each country are presented in the Additional file 1: Table S3. We received high response rates (>90%) in Cambodia and Vietnam, and a relatively high response (86% and 70% for district and hospital questionnaires, respectively) in Lao PDR. The response rates in Thailand were somewhat lower, especially for the hospital questionnaire with replies from approximately half of all the hospitals. (We suspect this may be a result of having previously conducted a resource survey as part of a pilot study in Thailand in 2007, which may have caused an element of fatigue among respondents in Thailand.) Maps of the resource data at district level, before extrapolation of missing data and pooling of data at province level, are given in Additional file 1: Figure S1. The results presented in the following sections are based on the availability of resources after extrapolation of missing data using the methods described earlier.

### Resource availability

**Figure 1:**
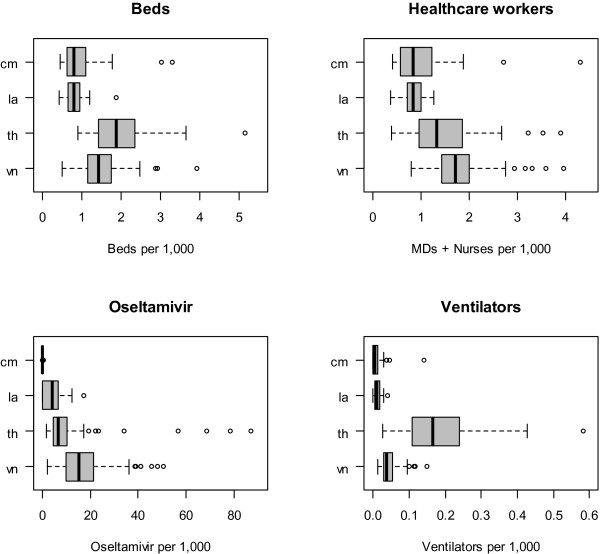
**Availability (per population) of healthcare resources for responding to pandemic influenza across provinces in four countries of the Greater Mekong Subregion.** Boxes represent the median and interquartile range, and whiskers represent the most extreme values within 1.5 times of the interquartile range. cm = Cambodia; la = Lao PDR; th = Thailand; vn = vietnam; HRH = human resources for health (medical doctors and nurses).

The statistical distributions of the density (number per capita) of the four resource items across provinces within each country are presented as box plots in Figure 1. The density of most resources showed substantial variation across provinces, with median values tending to be lowest in Cambodia and Lao PDR. The availability of ventilators was generally higher, although with very wide variation, across provinces in Thailand compared to the other three countries. Oseltamivir density showed a different pattern than the other three resources with high variation across provinces in all countries except Cambodia, where most provinces had no oseltamivir.

### Resource shortages

**Figure 2:**
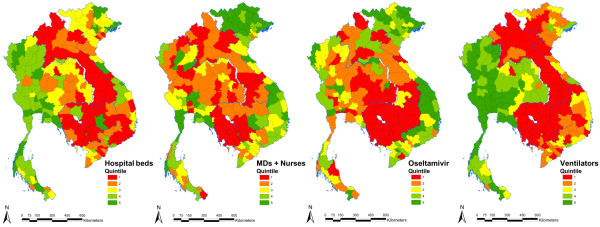
**Geographic distribution of healthcare resources (adjusted for population size) for responding to pandemic influenza across provinces in four countries of the Greater Mekong Subregion.** Relatively underserved and over-supplied provinces are operationally defined as those in the lowest and highest quintiles, respectively.

Underserved areas are operationally classified as those areas where resource densities fall in the lowest quintile of resource distribution based on all GMS provinces (Table 1 and Figure 2). Within the GMS, Cambodia and Lao PDR were the two countries with most provinces having relatively low resource densities. More than half of all provinces in Cambodia and Lao PDR were regionally underserved for health workers, ventilators, and hospital beds (Figure 2). Additionally, all Cambodian provinces were regionally underserved areas for oseltamivir. In Thailand, the northeastern provinces, and some southern and northern provinces, tended to have lower resource densities, particularly when compared with central provinces. In Vietnam, the southern and the northern mountainous provinces had lower densities of hospital beds and ventilators than other regions.

**Table 1:** Number of provinces that are relatively underserved for each resource, and their populations

	**Total Population (million)**	**Number Provinces**	**Number of provinces underserved for each resource type**				**% of population living in underserved provinces**			
			**Health workers**	**Beds**	**Ven-tilators**	**Osel-tamivir**	**Health workers**	**Beds**	**Ven-tilators**	**Osel-tamivir**
Cambodia	13.4	24	14	17	20	24	78	76	84	100
Lao PDR	5.6	17	9	14	15	7	66	76	90	27
Thailand	63.4	76	15	3	1	15	28	4	1	27
Vietnam	85.4	63	1	12	12	2	2	20	20	3

Underserved provinces are operationally defined as those with resource densities in the lowest quintile across all provinces.

In terms of the human population distribution across provinces, at least three quarters of the Cambodian population and two thirds of the Laotian population live in regionally underserved areas in relation to health workers, ventilators, and hospital beds. More than one quarter of the Thai population, living in 15 out of 76 provinces, are underserved for health workers and oseltamivir. Approximately one fifth of the Vietnamese population, living in 12 of the 63 provinces, is underserved for beds and ventilators.

Provinces with multiple shortages of more than one resource item were also identified (Table 2). Resource shortages/underserved provinces were more concentrated in Cambodia, where all 24 provinces were underserved for at least one resource, and 11 out of 24 provinces (with 65 percent of the total population) were regionally underserved for all four key resource items. In Lao PDR, while fewer provinces (3 out of 17, or 11 percent of Laotians) were underserved for all four resource items, all provinces were underserved for at least two resources. In Thailand and Vietnam, 37 and 40 percent of the population, respectively, live in provinces underserved by at least one resource item. However, no provinces in Thailand or Vietnam were underserved for all four items (Table 2).

**Table 2:** Number of provinces underserved by multiple resource items, and their populations

	**Total Population (million)**	**No. of provinces**	No. of provinces underserved* for 1, 2, 3 or 4 resource items				% of population living in provinces underserved* for 1, 2, 3, or 4 resource items			
			1	2	3	4	1	2	3	4
Cambodia	13.4	24	2	4	7	11	9	9	17	65
Lao PDR	5.6	17	0	7	7	3	7	37	45	11
Thailand	63.4	76	19	7	2	0	37	7	3	0
Vietnam	85.4	63	17	8	0	0	27	9	0	0

*Underserved provinces are operationally defined as those with resource densities in the lowest quintile across all provinces.

### Mismatch of resources

Across the GMS, there were 16 provinces with resource mismatches, that is, a relative “oversupply” of at least one resource item together with an undersupply of at least one other resource (Table 3). (We define oversupply as a province having a resource density in the top quintile in relation to other provinces in the GMS.) Most of these provinces were in Thailand and in Vietnam (seven each). No province in Lao PDR was observed to have a resource mismatch. In terms of the population, the issue was most pronounced in Thailand with approximately one fifth of the Thai population living in provinces with resource mismatches.

**Table 3:** Number of provinces with resource mismatches and their populations

	**Total Population (million)**	**No. of Provinces**	No. of provinces with a resource mismatch*	% of population living in provinces with resource mismatch*	**Population living in provinces with resource mismatch (million)**
Cambodia	13.4	24	2	10	1.3
Lao PDR	5.6	17	0	0	0
Thailand	63.4	76	7	21	13.2
Vietnam	85.4	63	7	6	5.4

*A resource mismatch is defined as a province that is both relatively underserved (lowest quintile) for at least one resource item and relatively oversupplied (highest quintile) for at least one other resource item.

### Spatial clustering and outliers of resource densities

**Figure 3:**
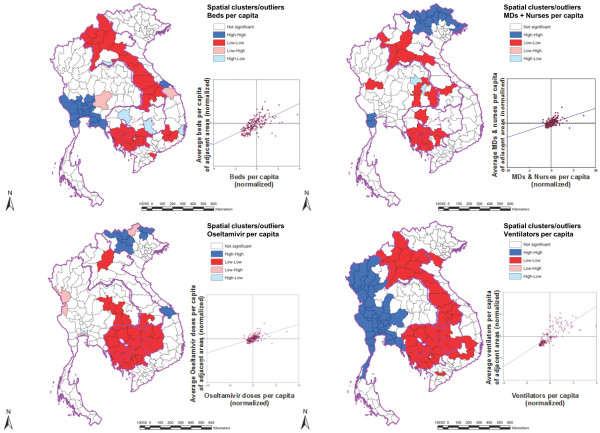
**Spatial clusters and outliers of healthcare resources across provinces in the Greater Mekong Subregion.** “High-high” and “low-low” clusters define a group of adjacent provinces with similarly high and low resource densities, respectively. A“high-low” cluster defines a province with relatively high resource density that is adjacent to provinces with relatively low resource densities, while a “low-high” cluster defines the converse pattern.

Spatial analysis allows us to identify patterns of geographic clustering of resource densities among provinces. As shown in Figure 3, the areas of high-high clustering in the GMS lie mainly in central Thailand and northern Vietnam. There were high-high clusters of hospital beds in central Thailand, health workforce and oseltamivir in northern Vietnam, and ventilators in central and northern Thailand.

Almost all provinces in Lao PDR, and western provinces of Cambodia, formed areas of low-low clustering for hospital beds. Low-low clusters of ventilators were located throughout most of Lao PDR and Cambodia, and some Vietnamese provinces. Oseltamivir low-low clusters were also mainly located in Cambodia, along with a few border provinces of Thailand (Figure 3).

Spatial outliers with regard to resource density across the region were also identified. For hospital beds, there were four high-low outliers (two in Cambodia, and one each in Vietnam and Lao PDR), and two low-high outliers (one each in Thailand and Vietnam). For health workforce, there were two high-low outliers in Thailand. For oseltamivir, there were four low-high outliers (three in Thailand and one in Vietnam); and one high-low outlier in Thailand. No spatial outliers were observed for ventilators.

**Figure 4:**
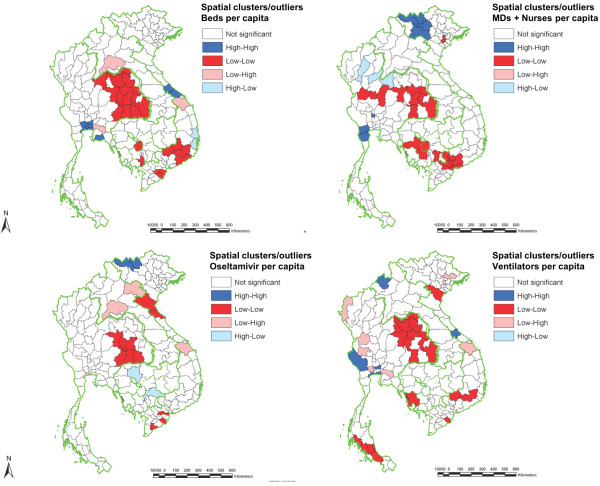
**Spatial clusters and outliers of healthcare resources across provinces within each country in the Greater Mekong Subregion.** In this analysis, resource densities are only compared among provinces within the same country (unlike in Figure 3). “High-high” and “low-low” clusters define a group of adjacent provinces with similarly high and low resource densities, respectively. A“high-low” cluster defines a province with relatively high resource density that is adjacent to provinces with relatively low resource densities, while a “low-high” cluster defines the converse pattern.

Spatial autocorrelation of health system resources was also calculated across provinces within each country individually (i.e., when resource density distributions were compared only across provinces within the same country) (Figure 4). The patterns were quite different from the overall GMS regional pattern. Lao PDR had the fewest spatial clusters or outliers, with one low-high outlier province for hospital beds, two low-high outlier provinces for oseltamivir, and one high-low outlier province for ventilators. Cambodia had a number of low-low clusters for hospital beds, health workforce, and ventilators, and two high-low outlier provinces for oseltamivir. Vietnam had all types of clusters and outliers for hospital beds and ventilators. For health workers, there was high-high clustering in the northwestern provinces and low-low clustering in the southern part of the country. There was also a mixture of patterns of spatial autocorrelation in the case of oseltamivir. Thailand showed low-low clustering for all four resource items in the northeast region, and also for ventilators in the south. In contrast, the central Thai provinces showed high-high clustering for all resource types except oseltamivir. For outliers, there was a number of low-high outliers for ventilators and hospital beds and a few high-low outliers for the health workforce in the northern Thai provinces.

### Summary index of inequality in resource distribution

In addition to spatial analysis, summary statistics of inequality in resource distribution across the GMS were calculated for each resource item (Table 4). Values for both the Gini coefficient and Theil index suggest that, across the GMS, the distribution of ventilators (Gini [*G*] = 0.53; Theil-T [*T*_T_] = 0.48) and oseltamivir (*G* = 0.50; *T*_T_ = 0.47) were highly unequal. Distributions of beds (*G* = 0.273; *T*_T_ = 0.13) and health workers (*G* = 0.24; *T*_T_ = 0.09) were somewhat more equal. Decomposition of the Theil-T Index is also useful in decomposing inequality to the component resulting from within-country inequality (between provinces) and the component from between-country inequality. For distribution of beds, health workers, and oseltamivir, inequalities across provinces within countries accounted for the majority of the Theil-T values. For ventilators, however, unequal distribution between countries accounted for slightly more of the total inequality than that within countries (Table 4).

**Table 4:** Summary indices of inequality in resource distribution across provinces in the Greater Mekong Subregion

**Type of resource**	**Gini Coefficient**	**Theil-T Index**		
		**Total**	**Within countries**	**Between countries**
Health Workers	0.236	0.093	0.071	0.021
Beds	0.273	0.126	0.102	0.024
Ventilators	0.525	0.482	0.219	0.262
Oseltamivir	0.504	0.466	0.378	0.089

Looking at inequality indices within each country (Table 5), ventilators were the most unequally distributed resource in all countries except Thailand, where oseltamivir had the most unequal distribution. For all resource items, inequality indices tended to be higher in Cambodia compared with other countries. Again, the exception was for oseltamivir, which was most unequally distributed within Thailand. Of the four countries, Vietnam tended to have the most equal resource distribution.

**Table 5:** Inequality in distribution of key resources across provinces within countries in the Greater Mekong Subregion

**Gini Coefficient**	**Health Workers**	**Beds**	**Ventilators**	**Oseltamivir**
Cambodia	0.395	0.337	0.645	0.251
Lao PDR	0.152	0.240	0.343	0.319
Thailand	0.240	0.266	0.367	0.607
Vietnam	0.144	0.224	0.351	0.310
All 4 countries	0.236	0.273	0.525	0.504

## Discussion

Our results show profound geographic variation in the distribution and availability of health system resources both between and within countries of the Greater Mekong Subregion of Asia. These results reflect the high socio-economic diversity in Southeast Asia and, alongside the wide range of public health challenges in the region [2,25], echo suggestions that Southeast Asia represents a microcosm of global health [26]. We show, moreover, that inequalities in resource distribution in the region result principally from within country differences. For example, even though Lao PDR and Cambodia are both relatively low-income countries, they share very different health system resource distribution patterns. In Lao PDR, resources are more homogeneously and equitably distributed with relatively few clusters or mismatches. This is, perhaps, the result of centralised governance and planning systems and relatively limited donor investment [27]. Cambodia, by contrast, shows a greater degree of diversity in resource distribution, with some pockets of high resource density. Though a model of central planning exists, Cambodia has been a recipient of considerable donor investment, and activities of non-governmental organisations in some programmatic areas is substantial [28]. Whether these investments have resulted in some distortions in resource distribution is an area that is open to further research.

Overall, both Lao PDR and Cambodia have few resources relative to the region as a whole. But northeast Thailand, too, shows similarly poor availability of key health system resources, a not insignificant finding given it is home to about 35 percent of Thailand’s population. We found particularly wide variation in the availability of ventilators and oseltamivir in Thailand where, consistently with previous studies [29], our results suggest that there is an inequitable distribution of health system resources rather than simply an overall nationwide shortage.

Through determinations of resource distribution and highlighting relative shortages and distributional mismatches, new investments and re-distribution of resources offers policy makers the potential to correct inequalities in resources, and ultimately in health outcomes. Several of the resources we have mapped serve generic purposes, with oseltamivir being the only influenza-specific resource. Thus, improved distribution of resources has the potential to benefit public health outcomes beyond mitigation of pandemic influenza, and shortages or maldistribution may hinder control and mitigation efforts. But for public health to benefit effectively from improved distribution of resources, political, administrative and contextual hurdles may need to be overcome, including the mobilisation of resources across administrative boundaries [30]. Whilst drugs and ventilators may be readily re-distributed, other resources such as hospital beds and human resources may be more challenging. Our analysis suggests, however, that inequities, at least within countries if not regionally, are most profound for resources that would be most readily mobilised. National policy and strategies to address these discrepancies through better resource allocation across areas are needed. Stronger coordination of resource availability and use for pandemic responses is likely to be particularly important in more decentralized systems.

A number of other contextual challenges for optimum distribution and utilisation of healthcare resources were identified in our previous qualitative analysis of pandemic preparedness programmes in relation to national health systems in our four GMS study countries (along with Indonesia and Taiwan) [30]. These include the need for: (i) greater emphasis on strategies for pandemic mitigation (since planning in the region was found to focus overwhelmingly on early detection and containment); (ii) translation of existing plans into operations, particularly at sub-national administrative levels; and, (iii) greater national ownership of preparedness activities, particularly in low resource countries where external funding is prominent, to ensure that the allocation of pandemic-related investments is aligned with national systems and priorities [30].

The inequalities observed between, as well as within, countries in this study also highlight an important role for supranational mechanisms to mitigate the public health impact of future pandemics. Regional bodies and networks such as ASEAN and the MBDS could play a supportive role in providing evidence, such as findings from this study, to member countries to identify resource needs and discrepancies, and help pinpoint specific areas for cooperation. Supranational mechanisms could also help coordinate support from external funding agencies, to ensure it is directed towards the geographic areas most in need in order to address the existing gaps at the national and subnational levels.

A limitation of this study is that data were not available from all districts, with missing data an issue particularly for districts in Thailand. To address this problem, we extrapolated using country-specific mathematical models that acknowledged province characteristics and we validated our findings through comparison with aggregate national data collected separately from ministries of health questionnaires. Another limitation is that the analysis that could not extend to cover potential determinants of resource availability, such as level of economic development and relative political power, due to such data not being available at subnational levels. This could be an area of future research when disaggregate data on potential determinants of resource availability are available at province level.

In conclusion, we show that considerable diversity exists in the distribution of healthcare resources within and across countries of the GMS, and that much of the geographic inequalities result from within countries. We also identify substantial mismatches and clustering in the distribution of resources, which has the potential to reduce the public health benefits that may accrue from their use. Future investment towards reducing inequalities in the distribution of health system resources, and policies to facilitate sharing and mobilisation of resources across administrative boundaries (both intra- and internationally), are likely to be important for mitigating the impact of public health challenges such as pandemic influenza. These lessons and our results can be drawn upon by national policy makers and the international donor community to support investment choices.

## Competing interests

The authors declare that they have no competing interests.

## Authors’ contributions

PH coordinated data collection, analysed the data and drafted the manuscript. IC performed the GIS analyses and helped draft the manuscript. JWR contributed to project coordination, data analysis, and drafting the manuscript. ST, WP, BP, PNC and PS coordinated data collection, participated in resource characterisation, and helped interpret the results. RC conceived, designed, and coordinated the study, and helped draft the manuscript. All authors read and approved the manuscript.

## Supplementary Material

Additional file 1 Supplementary information. Click here for file
