# Supplementary material for: An analysis of health system resources in relation to pandemic response capacity in the Greater Mekong Subregion
Source: Int J Health Geogr. 2012 Dec 14;11:53. doi: 10.1186/1476-072X-11-53 (PMC3556110; doi:10.1186/1476-072X-11-53)

**Additional File: Supplementary Information**

**Table S1. List of health system resources required in pandemic response that were included in the questionnaires.** The list was derived from a systematic literature review, followed by a Delphi consensus process among a panel of 24 public health experts from Southeast Asia and Europe.

|  | ***Resource item*** |
| --- | --- |
| 1 | Hospital beds |
| 2 | Negative pressure room |
| 3 | Single occupancy room |
| 4 | ICU beds |
| 5 | Temporary clinical care beds |
|  | **Equipment** |
| 6 | Ambulance |
| 7 | Other transport vehicles |
| 8 | X-ray/ radiographic machine |
| 9 | Adult mechanical ventilator |
| 10 | Paediatric mechanical ventilator |
|  | **PPE and drugs** |
| 11 | N-95 / N-99 mask |
| 12 | Surgical mask |
| 13 | Face shield |
| 14 | Surgical gloves |
| 15 | Cover-all gown |
| 16 | Pneumococcal vaccine |
| 17 | Oseltamivir |
| 18 | Zanamivir |
| 19 | Amoxicillin |
| 20 | Co-trimoxazole |
| 21 | IV Fluid, 0.9% Normal Saline Solution |
| 22 | Body bag |
|  | **Laboratory** |
| 23 | Complete blood count |
| 24 | Serum bio-chemical testing |
| 25 | Bacterial culture and Drug sensitivity test |
| 26 | BSL-2 Laboratory |
| 27 | BSL-3 Laboratory |
| 28 | Conventional RT-PCR |
| 29 | Real-time RT-PCR |
| 30 | Oxygen |
|  | **Access to communication technology** |
| 31 | Telephone |
| 32 | Mobile Phone |
| 33 | Facsimile |
| 34 | Short-wave raido calls |
| 35 | Internet / Email |
|  | **Human resources** |
| 36 | Doctors |
| 37 | GPs |
| 38 | Internal med |
| 39 | Other doctors |
| 40 | Nurses |
| 41 | Pharmacists |
| 42 | Lab technicians |
| 43 | Public health personnel |
| 44 | Volunteer/community health workers |
| 45 | Administrative staff |
| 46 | Epidemiologist |
| 47 | Central Rapid response teams |
| 48 | Regional Rapid response teams |
| 49 | Province Rapid response teams |
| 50 | District Rapid response teams |
|  | **Health care facilities and their bed capacities** |
| 51 | Public tertiary/regional hospital |
| 52 | Public provincial/general/secondary level hospital |
| 53 | Public first level/community hospital |
| 54 | Other public hospital |
| 55 | Private hospital |
| 56 | Health centre/primary care unit |
| 57 | Drug store/pharmacy |

**Text S1. Data extrapolation methods**

We used linear prediction models to extrapolate the missing values based on a number of district characteristics such as total number of hospital beds or public hospital beds, population size, geographic location (region/province). The extrapolation exercise was done separately for each resource and for each country to obtain best model fit. Specifically,

- For doctors and nurses, missing values were extrapolated based on total number of beds in the district (disaggregated by public and private beds if available) except in Lao PDR where total population was used instead. The extrapolation also took into account geographical aspect by including province or regional dummy variables in the models.
- For oseltamivir, extrapolation exercise was done separately for drug availability at hospital level and at district health office level. In Cambodia and Lao PDR, the average value of zero was used as nearly all hospitals and districts for which data were available had no oseltamivir. In Thailand, a two step model was used: first to predict the likelihood of having antiviral stockpile and second to predict the number of stockpile. The model relies on the district population or total number of beds and geographical location.
- We used similar methods to oseltamivir availability extrapolation for adult and pediatric ventilators.

**Table S2.** Variables used in linear prediction models for extrapolating missing data across districts in each country.

|  | **Bed** | **Doctors** | **Nurses** | **Oseltamivir in Hospital settings** | **Oseltamivir at District Offices** | **Ventilators** |
| --- | --- | --- | --- | --- | --- | --- |
| Cambodia |  |  |  | mean (0) | mean (0) |  |
| Lao PDR |  | pop, prov | pop, prov | mean (0) | mean (0) |  |
| Thailand |  | pub/prv, bed, reg | pub/prv, bed reg | 2s, bed, reg | 2s, bed, type, reg | 2s, bed, type, reg |
| Vietnam |  | pub/prv, bed, | pub/prv, bed | 2s, bed | 2s, bed |  |

pop = population size; bed = hospital bed; prov = province; reg = region; type = facility type; pub/prv = public/private; 2s = two-stage model

**Table S3.** Data collection and response rates.

|  | District questionnaire | | |  | Hospital Questionnaire | | |
| --- | --- | --- | --- | --- | --- | --- | --- |
|  | Number of Districts | Questionnaires Returned | Response rate (%) |  | Number of Hospitals | Questionnaires Returned | Response rate (%) |
| Cambodia | 77* | 77 | 100 |  | 185 | 185 | 100 |
| Lao PDR | 141 | 121 | 86 |  | 161 | 112 | 70 |
| Thailand | 927 | 547 | 59 |  | 1279 | 603 | 47 |
| Viet Nam | 658 | 630 | 96 |  | 873 | 813 | 93 |

*****Districts of Cambodia represent the Ministry of Health’s Operational Districts.

**Figure S1.** Geographic distribution of selected healthcare resources for responding to pandemic influenza across districts in four countries in the Greater Mekong Subregion.


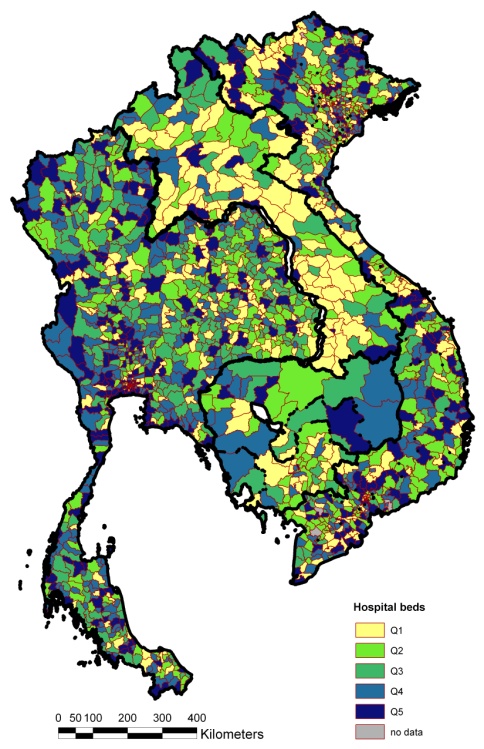

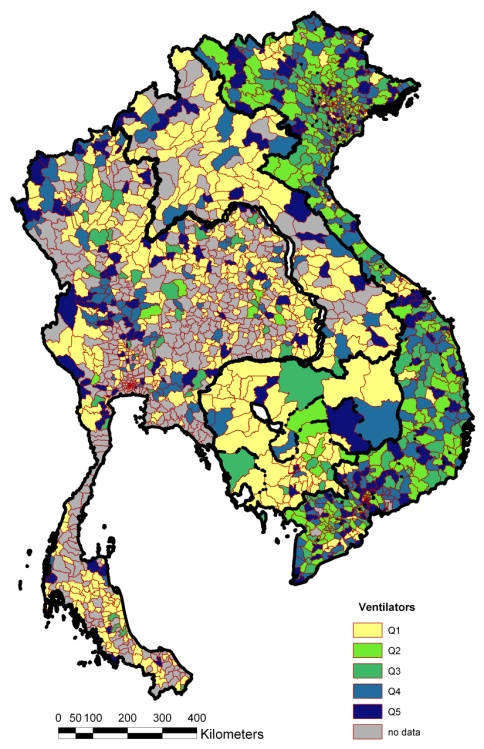


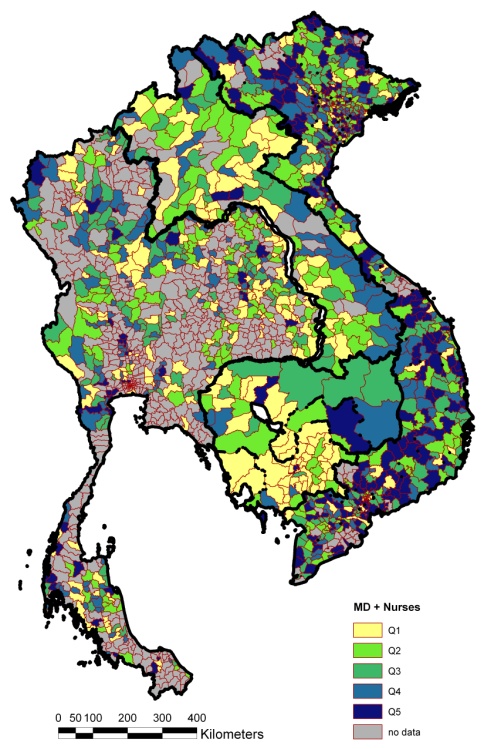

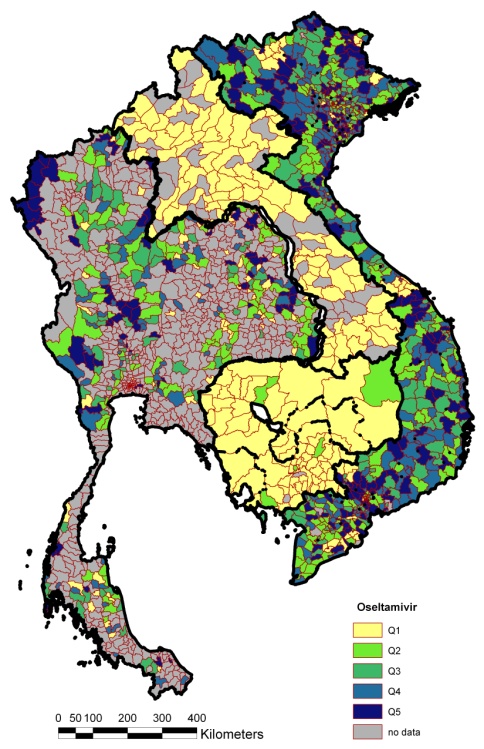

Supplement: Additional file 1 — Supplementary information. [file 1476-072X-11-53-S1.docx]
